# Four new species of *Oreocharis* (Gesneriaceae) in Yunnan province, China

**DOI:** 10.3897/phytokeys.157.32284

**Published:** 2020-08-26

**Authors:** Wen-Hong Chen, Ya-Mei Zhang, De-Ming He, Yong-Liang Li, Yu-Min Shui

**Affiliations:** 1 CAS Key Laboratory for Plant Diversity and Biogeography of East Asia, Kunming Institute of Botany, Chinese Academy of Sciences, 132 Lanhei Road, Kunming 650201, Yunnan, China; 2 Karst Conservation Initiative of Yunnan, Kunming 650201, Yunnan, China; 3 University of Chinese Academy of Sciences, Beijing 100049, China; 4 The Management Bureau of Wenshan National Nature Reserve, Wenshan 663000, Yunnan, China; 5 Forestry Bureau of Yongde County, Yongde 677600, Yunnan, China

**Keywords:** Flora of Yunnan, Montane forests, New species, South-western China, Subtropic regions, Yellow flowers

## Abstract

Four new species of *Oreocharis* (Gesneriaceae) are described and illustrated. These new species grow in pairs in montane forests in Yunnan province, China. One pair grows in Wenshan county, Southeast Yunnan, viz. *Oreocharis eriocarpa* W.H. Chen & Y.M. Shui and *O. wenshanensis* W.H. Chen & Y.M. Shui and another pair grows in Yongde county, Southwest Yunnan, viz. *O. fulva* W.H. Chen & Y.M. Shui and *O. lacerata* W.H. Chen & Y.M. Shui. Their morphological and geographical relationship with similar species is discussed and the IUCN endangered status is provided, based on the available data.

## Introduction

**Figure 1.**
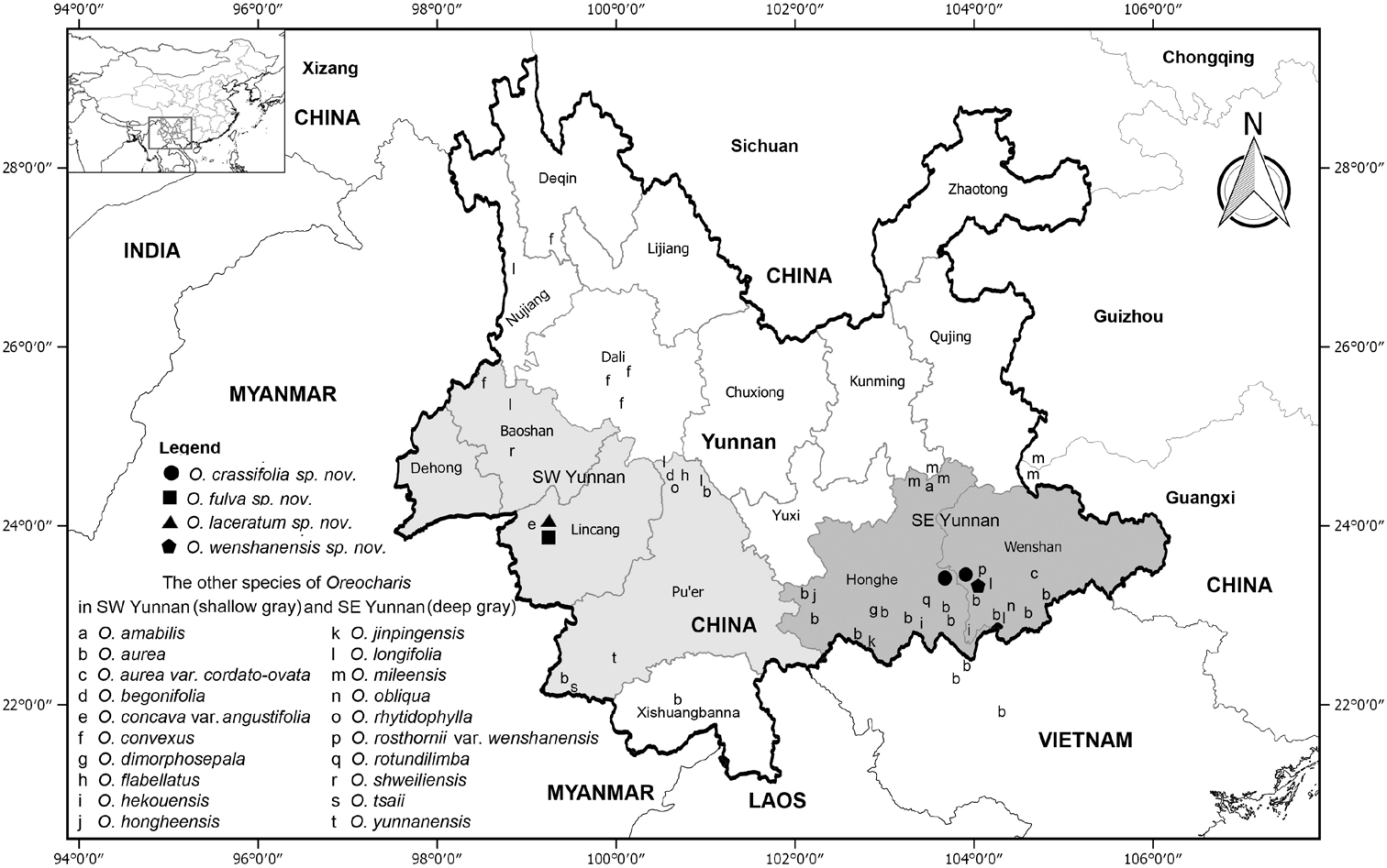
The localities of the four new species and the other species of *Oreochariss.l.* from SW Yunnan and SE Yunnan in SW China. The districts are shown in Yunnan province, SW China.

**Figure 2.**
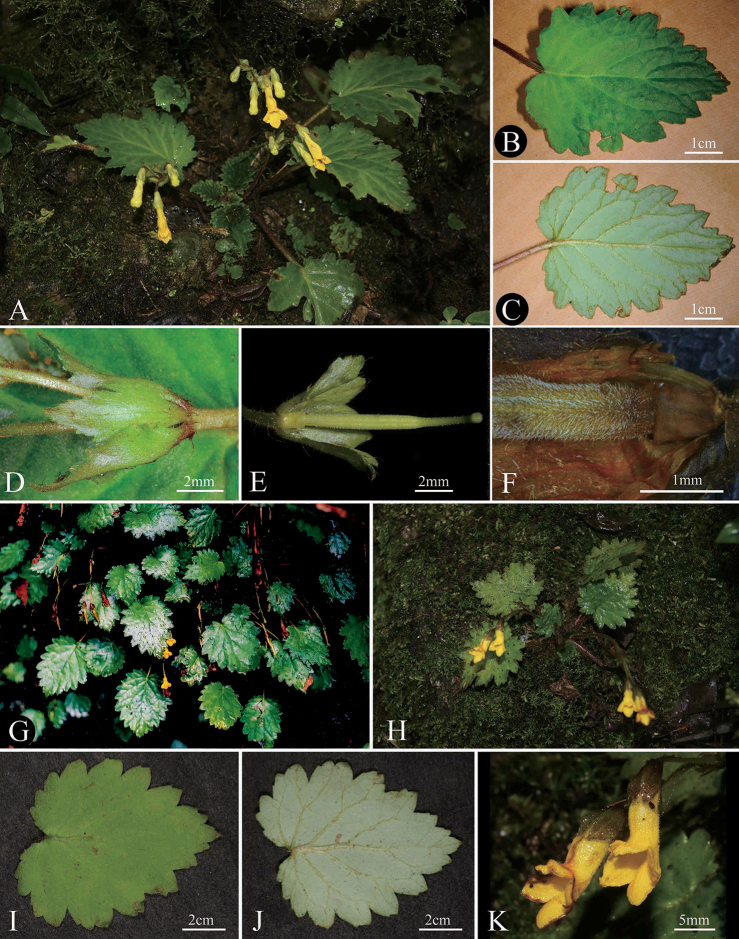
*Oreocharis eriocarpa* W.H.Chen & Y.M.Shui, sp.nov. (**A–F**) **A** habit **B** leaf adaxial surface **C** leaf abaxial surface **D** bracts **E** ovary and disc **F** dense pubescent ovary and disc; *O. wenshanensis* (**G–K**) **G** habitat **H** plant **I** leaf adaxial surface **J** leaf abaxial surface **K** flower. Photography by He De-Ming (**A–E, K**), Zhang Ting (**E**), Chen Li (**F**), Shui Yu-Min (**G, I, J**).

**Figure 3.**
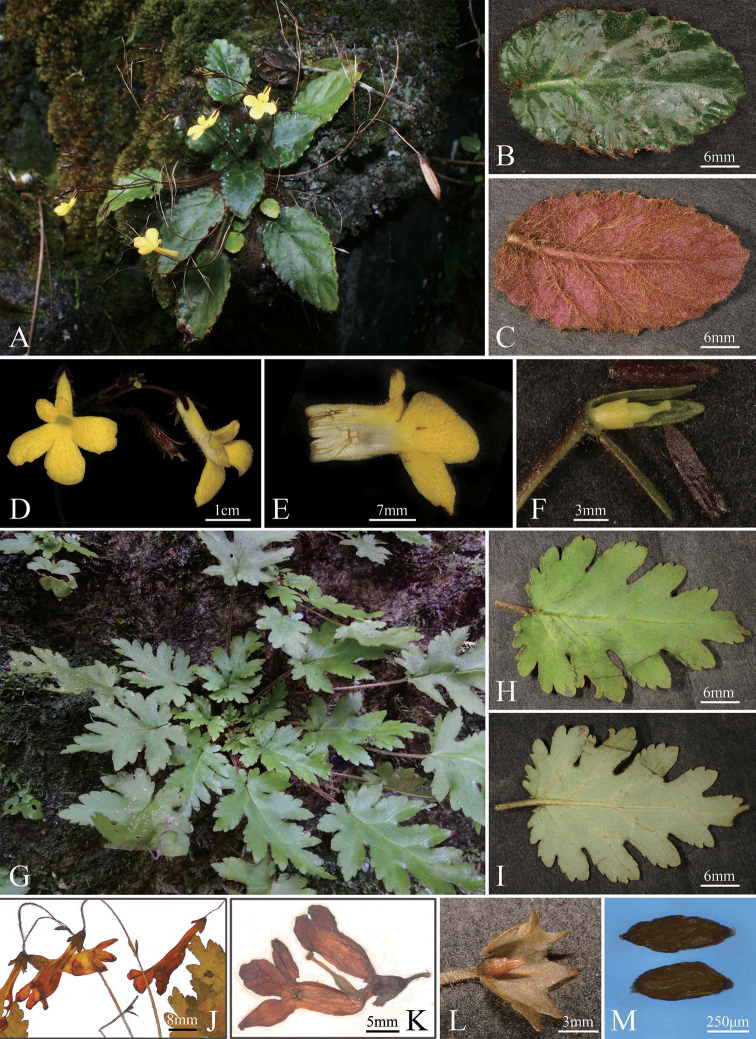
*Oreocharis fulva* W.H.Chen & Y.M.Shui, sp.nov. (**A–F**) **A** habitat **B** leaf abaxial surface **C** leaf abaxial surface **D** flower **E** open corolla **F** calyx and ovary; *O. lacerata* (**G–M**) **G** plant **H** leaf adaxial surface **I** leaf abaxial surface **J** lateral view of flower **K** lateral opened flower **L** disc and ovary **M** seeds. Photography by Li Yong-Liang (**A, G**), Shui Yu-Min (**B–G, H, I, L**), Chen Li (**J–M**).

In China, Southeast Yunnan and Southwest Yunnan are rich in species diversity of the genus *Oreocharis* s.l. (Gesneriaceae) (Fig. 1, Li and Wang 2005, Möller et al. 2011). Firstly, bordering North Myanmar, SW Yunnan includes Baoshan, Dehong Dai and Jingpo Autonomous Prefecture, Lincang and Pu’er districts with 11 species of the genus, viz. *O. begoniifolia* (H.W.Li) Mich.Möller & A.Weber, O. concava (Craib) Mich.Möller & A.Weber var. angustifolia (K.Y.Pan) Mich.Möller & A.Weber, *O. convexa* (Craib) Mich.Möller & A.Weber, *O. flabellata* (C.Y.Wu ex H.W.Li) Mich.Möller & A.Weber, *O. longifolia* (Craib) Mich.Möller & A.Weber, *O. shweliensis* Mich.Möller & W.H.Chen, *O. rhytidophylla* C.Y.Wu ex H.W.Li, *O. tsaii* Y.H.Tan & Jian W.Li and *O. yunnanensis* Rossini & J.Freitas (Li and Wang 2005, Möller et al. 2011, Tan et al. 2013, Rossini and Freitas 2014, Tan et al. 2015). Amongst them, O. concava var. angustifolia, *O. flabellata*, *O. begoniifolia*, *O. rhytidophylla*, *O. tsaii* and *O. yunnanensis* are endemic to SW Yunnan. Amongst the two species without morphology of flowers in the protologue (Li 1983), *O. rhytidophylla* has been supplemented (Zhang et al. 2019) and *O. flabellata* has still not been confirmed up to now. Secondly, bordering Vietnam, SE Yunnan includes Honghe Hani and Yi Autonomous Prefecture and Wenshan Zhuang and Miao Autonomous Prefecture with 10 species of the genus, viz. *O. amabilis* Dunn, *O. aurea* Dunn, O. aurea var. cordato-ovata C. Y. Wu ex H. W. Li, *Oreocharis dimorphosepala* (W. H. Chen & Y. M. Shui) Mich. Möller, *O. hongheensis* W.H. Chen & Y.M. Shui, *O. jinpingensis* W.H. Chen & Y.M. Shui, *O. mileensis* (W.T.Wang) Mich.Möller & A.Weber, *O. obliqua* C. Y. Wu ex H. W. Li, O. rosthornii (Diels) Mich. Möller & A.Weber var. wenshanensis (K.Y.Pan) Mich. Möller & A.Weber and *O. rotundifolia* K. Y. Pan. Amongst these *O. amabilis*, O. aurea var. cordato-ovata, *O. dimorphosepala*, *O. hongheensis*, *O. jinpingensis*, *O. obliqua*, O. rosthornii var. wenshanensis and *O. rotundifolia* are endemic to SE Yunnan (Li and Wang 2005, Möller et al. 2011, Chen et al. 2012, 2013, 2017, Cai et al. 2015). Nevertheless, the recent exploration reveals that there are an additional four new species needing to be described in the genus from Yongde county of Lincang District in SW Yunnan and Wenshan county of Wenshan Zhuang and Miao Autonomous Prefecture in SE Yunnan (Figs 2, 3). The new findings complement the species richness of the genus in the above regions in China (Fig. 1; Wang et al. 1990, Wang et al. 1998 onwards, Li and Wang 2005, Liu and Peng 2010, Shui and Chen 2010).

The four new species have been recently confirmed, based on the morphological evidence instead of molecular data in the expanded genus. Firstly, the recent combined analysis of ITS and *trn*L-F revealed the possible rapid radiation and low resolution of the phylogenetic trees (Möller et al. 2011, Chen et al. 2014), implying that the molecular data from few molecular makers just provide affinity between similar endemic species rather than whether or not they are conspecific. Therefore, more molecular makers, transcriptome or genome data will need to be adopted to resolve the above question. Secondly, based on the recent phylogenetic study, the genus seems to be divided into two groups, which are dominated respectively by diandrous or tetrandrous flowers with purple flowers, south-eastern China distribution and usually less than 1600 m elevation and by tetrandrous flowers with yellow flowers, south-western China and usually more than 2000 m elevation (Möller et al. 2011, Chen et al. 2014, Zhang et al. 2018). Thus, the four new species which we proposed should be a member of the second group because of the tetrandrous, yellow-flowered and more than 2000 m elevation. Thirdly, amongst the second group, we consulted the actual specimens and on-line images in the important herbaria in China (KUN, PE) and worldwide (BM, E, K, P) and confirmed the potential similarity of the new species we proposed. Furthermore, due to the high endemism in the genus, we paid more attention to the species growing in the same regions and designed an identification key to differentiate the new species and the other species of the two species groups, respectively from SW Yunnan and SE Yunnan, China. Finally, we provided both the tables showing the differences between the new species and the most similar species, as well as colour figures showing their detailed and actual morphology besides their illustration.

## Key to the species of *Oreocharis* from SW Yunnan and SE Yunnan, China

**Table:** 

1	Flower purple	**2**
–	Flower yellow or orange	**7**
2	Leaf blade round, base cordate	**3**
–	Leaf blade lanceolate or elliptic, base cuneate	**4**
3	Stamens exserted (SW Yunnan)	***O. begoniifolia***
–	Stamens included (SE Yunnan)	***O. dimorphosepala***
4	Leaf blade lanceolate, acuminate on the top (SE Yunnan)	**5**
–	Leaf blade elliptic, obtuse on the top (SE Yunnan)	***O. jinpingensis***
5	Corolla tube-form, ovary and fruit glabrous (SE Yunnan)	***O. obliqua***
–	Corolla narrowly campanulate, ovary and fruit pubescent	**6**
6	Leaf blade surfaces white pubescent, staminode 1–2.2 mm (SE Yunnan)	**O. rosthornii var. wenshanensis**
–	Leaf blade rust-brown villous, staminode 2.5–3 mm (SW Yunnan)	***O. shweliensis***
7	Corolla narrowly campanulate, yellow with purple dots inside (SE and SW Yunnan)	***O. longifolia***
–	Corolla tubiformis, yellow without purple dots inside	**8**
8	Ovary and fruit pubescent	**9**
-	Ovary and fruit glabrous	**10**
9	Leaf blade ovate, base cordate (SW Yunnan)	***O. eriocarpa* sp. nov.**
–	Leaf blade narrowly elliptic, base cuneate (SW Yunnan)	**O. concava var. angustifolia**
10	Leaf blade broadly ovate or ovate, base cordate	**11**
–	Leaf blade elliptic or lanceolate, base cuneate or shallow cordate	**13**
11	Corolla tube constricted at throat	**12**
–	Corolla tube not constricted at throat (SW Yunnan)	***O. yunnanensis***
12	Calyx lobes more than 1/2 longer than corolla tube (SE Yunnan)	***O. rotundifolia***
–	Calyx lobes less than 1/5 longer than corolla tube (SW Yunnan)	***O. tsaii***
13	Stamens not exserted	**14**
–	Stamens exserted (SE Yunnan)	***O. hongheensis***
14	Calyx connate	**15**
–	Calyx free	**18**
15	Leaf blade lobed up to 1/3 (SW Yunnan)	***O. lacerata* sp. nov.**
–	Leaf blade not lobed	**16**
16	Adaxial corolla lips emarginate to undivided	**17**
–	Adaxial corolla lips 2-lobed (SE Yunnan)	***O. wenshanensis* sp. nov.**
17	Filaments glabrous, disc 2–5 mm, 5-lobed (SW Yunnan)	**O. concava var. concava**
–	Filaments sparsely puberulent, disc ca. 1 mm, entire (SW Yunnan)	***O. convexa***
18	Abaxial lips 2-lobed, adaxial lips 3-lobed	**19**
–	Abaxial lip emarginate to undivided, adaxial lips 4-lobed (SW Yunnan)	***O. mileensis* or *O. amabilis***
19	Leaf blade apex acute	**20**
–	Leaf blade apex retuse	**21**
20	Filaments pubescent, staminode ca. 0.5 mm (SE and SW Yunnan)	**O. aurea var. aurea**
–	Filaments white villous, staminode ca. 2 mm (SE Yunnan)	**O. aurea var. cordato-ovata**
21	Plants golden-brown villous (SW Yunnan)	***O. fulva* sp. nov.**
–	Plants dense brown pubescent	**22**
22	Leaf blade adaxially sparsely villous (SE Yunnan)	***O. hekouensis***
–	Leaf blade adaxially glabrous (SW Yunnan)	***O. rhytidophylla***

The above new discovery depended on the long-term field exploration from the local forestry staff. In general, most of the species in *Oreocharis* are prone to grow on the north-facing shady slope nearby the summit, especially in SW China (Li and Wang 2005) and so it is difficult to find them in the field except in inaccessible localities. For example, as for *Orocharis wenshanensis* W.H.Chen & Y.M.Shui in the core region of Wenshan Laojunshan National Nature Reserve, the staff (DMH in authorship) of the Natural Reserve had searched for it for several years since 2008 and found it in 2013 even if the preliminary record was from the previous intergraded surveys (Shui et al. 2008). Another example is from the staff member (YLL) of the forestry department of Yongde county. He also went to the core regions of Yongde Daxueshan National Nature Reserve to search for it in 2013 even if the information on record was from the previous intergraded surveys in 2003 (Liu and Peng 2010). Therefore, during the exploration of the genus, the local staff provided considerable contributions to the new discovery for science and to the conservation of the regional biodiversity.

## Taxonomy

### Oreocharis eriocarpa

Taxon classificationPlantaeLamialesGesneriaceae

W.H.Chen & Y.M.Shui sp. nov.

E0D86650-B9FF-5EAF-8EEC-4ED76AEAA8CA

urn:lsid:ipni.org:names:77211183-1

Fig. 4

#### Diagnosis.

The new species is similar to *Oreocharis concava* (Craib) Mich.Möller & A.Weber, but different in broadly ovate bracts (*vs.* narrowly oblong to obovate), corolla strigose outside (*vs.* pubescent), calyx 5-sect up to 1/3 from base (*vs.* above middle) and ovary and fruit pubescent (*vs.* glabrous).

**Figure 4.**
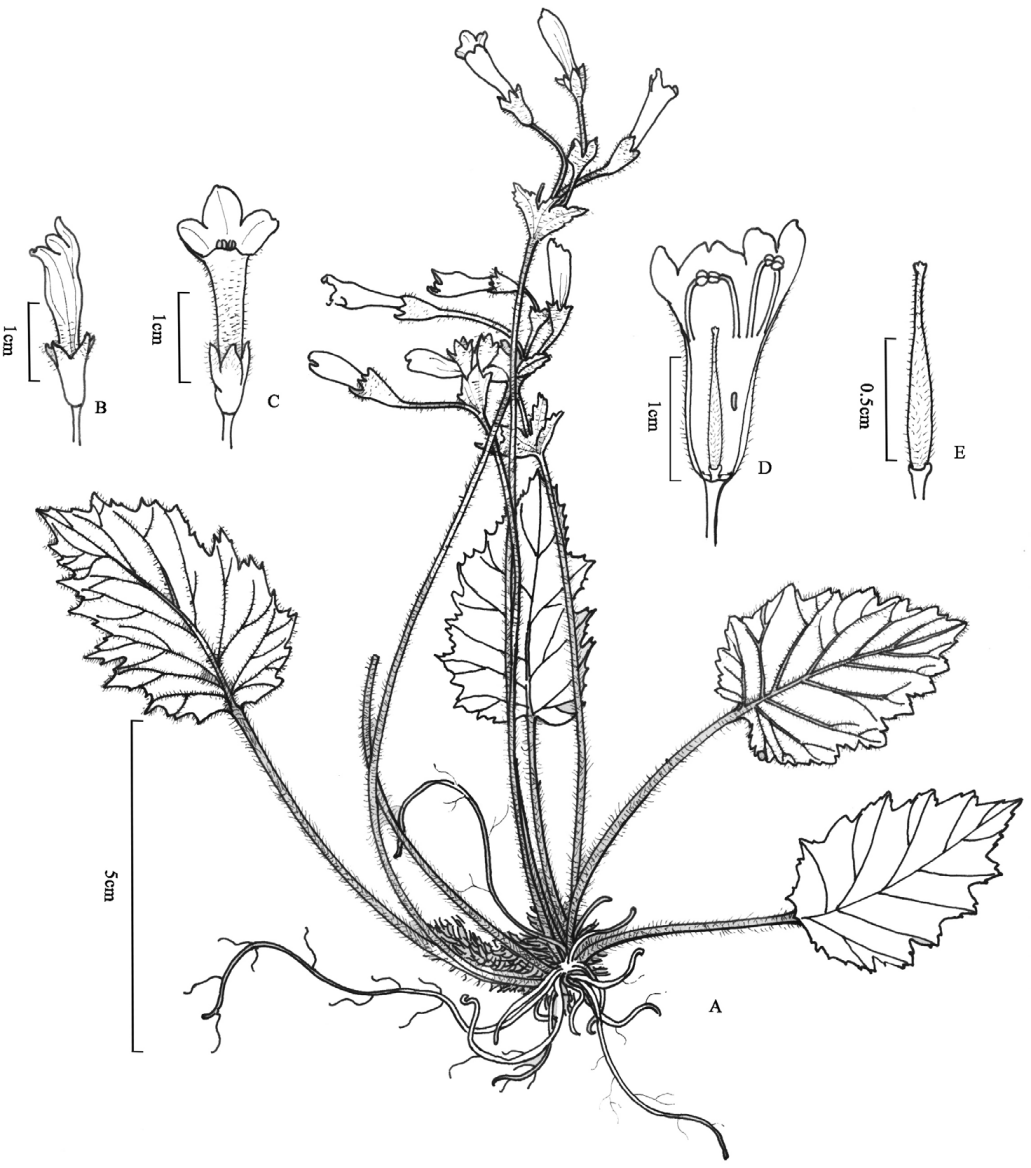
*Oreocharis eriocarpa* W.H.Chen & Y.M.Shui, sp. nov. **A** plant **B** lateral view of flower **C** frontal view of flower **D** opened corolla showing stamens, anthers, disc and staminode **E** pistil showing ovary, disc and stigma. (Drawn by Ling Wang from holotype).

#### Type.

CHINA. Yunnan Province: Wenshan Zhuang and Miao Autonomous Prefecture, Wenshan county, Laohuilong community, Laowuji village, 103°51'13.17"E, 23°20'29.35"N, alt. 2168 m, on rocks in the forests, 30 July 2013, *Shui Y.M., He D.M. et al. B2013-304* (holotype, KUN; isotype, PE).

#### Description.

Herb perennial and stemless, rhizomatous. Leaves basal; petiole 4–8 cm, densely brown villous; leaf blade ovate, thickly chartaceous, 4.0–5.2 × 3.0–3.5 cm, adaxially setulose and rugose, abaxially glabrous amongst veins, sparsely brown villous along veins, base cordate, apex acute, margin biserrate, lateral veins 5–6 on each side of midrib, indistinct adaxially and distinct abaxially. Inflorescences axillary; peduncle 9–13 cm, densely brown villous; bracts 2, leaf-like, broadly ovate, 7–10 × ca. 5 mm, adaxially glabrous and abaxially villous, margin serrate. Pedicel 1–2.5 cm, villous. Calyx ca. 6 mm, 5-sect from 1/3; segments equal, triangular, ca. 4 × 1.5–2 mm, margin entire below top, top crenate, adaxially glabrous, abaxially pubescent. Corolla yellow, 1.6–2.9 cm, outside strigose and inside glabrous; tube campanulate-cylindric, gradually slightly widening from middle of tube, 1.3–2 cm, 0.3–0.4 cm in diam., throat not constricted; limb 2-lipped; adaxial lip smaller, 3–5 mm, emarginate; abaxial lip larger, 3-lobed, lobes oblong, apex rounded, central lobe ovate, ca. 7 × 5 mm, lateral lobes rotund, ca. 5 × 5 mm. Stamens 4, coherent in 2 pairs, included, adaxial stamens 3–6 mm, adnate to corolla tube 7–13 mm from base, abaxial stamens 3–4 mm, adnate to corolla tube 13–16 mm from base; filaments tender, glabrous; anthers basifixed, oblong, 2-loculed, dehiscing longitudinally; staminode ca. 0.5 mm, adnate to corolla tube ca. 8 mm. Disc ring-like, 1–2 mm, 5-lobed shallowly. Pistil 0.9–1.8 cm, pubescent; ovary oblong, pubescent, 0.5–1.2 cm, 1-loculed; style glabrous, 0.4–0.6 cm; stigma 1, 2-lobed. Capsule straight, narrowly oblong, 3.4–4 cm × 0.8–0.9 cm, existing style ca. 0.7 cm. Seeds not seen.

#### Distribution, habitat and phenology.

The new species is endemic to SE Yunnan of China, on rocks or limestone cliffs. Flowering is July–September; and fruiting is October–January the following year.

#### Conservation status.

So far, there are two populations of the new species observed in the field (Fig. 1). One is in the type locality at the core position of the nature reserve with ca. 500 mature individuals and ca. 10, 000 m^2^ (100 m × 100 m) area, the other is the Pingbian county with ca. 120 mature individuals and ca. 1, 200 m^2^ (20 m × 60 m) area. According to the IUCN Red List Categories and Criteria, the new species is hereby assessed as “Vulnerable (VU)” (D1+D2). (IUCN 2012).

#### Additional specimens examined (paratype).

CHINA, Yunnan Province: Honghe Hani and Yi Autonomous Prefecture, Pingbian county, Heping community, Baige village,103°52'36.71"E, 23°17'24.68"N, 26 August 2015, *Shui Y.M. et al. B2015-315A* (KUN). Wenshan Zhuang and Miao Autonomous Prefecture: Wenshan county, Laohuilong community, Laowuji village, Matangqing, 103°51'14.33"E, 23°20'29.96"N, in fruits, 20 October, 2012, *De-Min He and Ting Zhang WSLJS558* (KUN); the same locality, 103°51'13.17"E, 23°20'29.35"N, on rocks in the forests, alt. 2168 m, in flower, 16 August 2018, *Ting Zhang, De-Min He and Yan-Fei Feng 18CS17589* (KUN).

#### Etymology.

The species is named after the pubescent fruits (Fig. 2F).

#### Note.

The new species is endemic to the border regions of Honghe Hani and Yi Autonomous Prefecture and Wenshan Zhuang and Miao Autonomous Prefecture in SE Yunnan (Fig. 1), at ca. 2100 m elevation. It is unique in its pubescent ovary and fruits (Fig. 2A–F). Its similar species (*Oreocharis concava*) is distributed in Northwest Yunnan at 2,800–3,600 m elevation, viz. Dali Bai Autonomous Prefecture, Lijiang District and Deqin Zang Autonomous Prefecture (Fig. 1). Table 1 shows the other differences between the above two species. Thus, there is an obvious geographical substitution between the above two species. Besides, after the examination of type specimens in PE and consulting the literature (Pan 1987, Li and Wang 2005, Möller et al. 2011), the new species seems similar to its another variety [Oreocharis concava var. angustifolia (K.Y.Pan) Mich.Möller & A.Weber] in the pubescent ovary, but obviously different from it in its ovate blade (*vs.* elliptic). It is possible that the latter variety might be a different species from the original variety and needs to be explored in the future.

**Table 1.** Differences in characters between *Oreocharis eriocarpa* and *O. concava* in Gesneriaceae.

Characters	*O. eriocarpa* sp.nov.	*O. concava*
leaves	thick-chartaceous	thin chartaceous
abaxial surface of leaf	glabrous amongst veins	densely white pubescent amongst veins
corolla	strigose outside	pubescent outside
bracts	broadly ovate, 7–10 × ca. 5 mm	narrowly oblong to obovate, 4–7 × 1–2 mm
calyx	ca. 6 mm, 5-sect up to 1/3 from base, lobes 3–4 mm long	7–10 mm, 5-sect up to 1/4 from middle, lobes 1–2.5 mm long
ovary	densely pubescent	glabrous
fruit	pubescent	glabrous
elevation	ca. 2100 m	3100–3600 m
distribution	Southeast Yunnan, China	Northwest Yunnan, China

### Oreocharis fulva

Taxon classificationPlantaeLamialesGesneriaceae

W.H.Chen & Y.M.Shui sp. nov.

5A17C2D4-84F6-53F7-9AF3-CE22EA3530C5

urn:lsid:ipni.org:names:77211184-1

Fig. 5

#### Diagnosis.

The new species is similar to *Oreocharis georgei* J. Anthony, but different in shallowly cordate leaf base (*vs.* narrowly cuneate), corolla ca. 2.4 cm long (*vs.* 1.4–1.9 cm), abaxial lip of corolla ca. 12 mm long (*vs.* 5–6 mm), the corolla throat not constricted (*vs.* constricted) and stamens coherent in 2 pairs (*vs.* separated).

**Figure 5.**
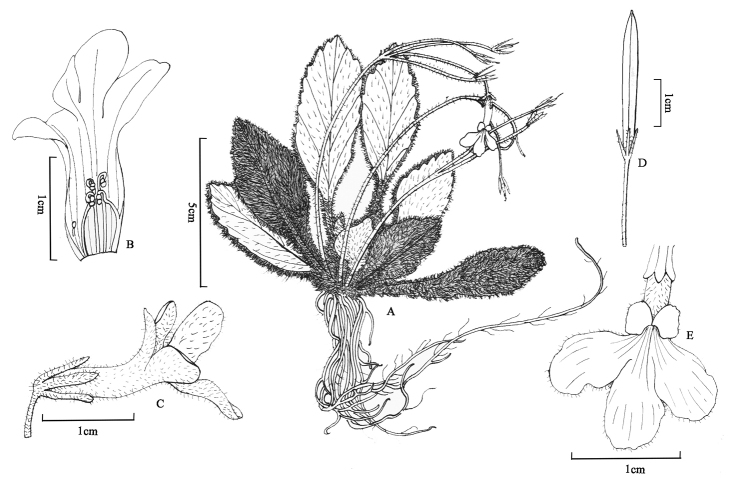
*Oreocharis fulva* W.H.Chen & Y.M.Shui, sp. nov. **A** plant **B** opened corolla showing stamens and staminode **C** lateral view of flower **D** fruit **E** face view of the corolla, showing the large abaxial limb lobes. (Drawn by Ling Wang from holotype).

#### Type.

CHINA. Yunnan Province: Lincang district, Yongde county, Daxue Mt., on rocks at forest margins along slope, 99°41'25"E, 24°11'50"N, elev. 2,000 m, 13 September 2013, in flower, *Shui Y.M. et al. B2013-579* (holotype, KUN; isotype, KUN, PE).

#### Description.

Herbs perennial, stemless, rhizomatous. Leaves basal. Petiole 1–3 cm long, golden-brown villous; leaf blade elliptic, thickly chartaceous or nearly leathery, 2.8–4 × 1.2–1.6 cm, adaxially green, sparsely long golden-brown villous, abaxially red-brown and with densely long golden multi-articulate hairs (especially on midrib), base shallowly cordate, apex obtuse, margin widely crenate and ciliate; lateral veins ca. 5 pairs on each side of midrib. Inflorescences axillary, multi-flowered. Peduncles 4.5–7 cm, golden-brown villous; bracts 2, very small, linear, ca. 6 × 1 mm. Calyx 5-sect to base, lobes lanceolate or linear, ca. 8 × 1 mm, adaxially green and glabrous, abaxially red-brown and golden-brown villous. Corolla yellow, ca. 2.4 cm long, outside white glandular pubescent and inside glabrous; corolla tube cylindrical, not constricted at throat, ca. 1.2 cm long, ca. 0.2 cm in diam., more or less curving; limb 2-lipped, adaxial lip 0.3–0.4 cm, 2-lobed, much shorter than abaxial lip, lobes oblong or subround, ca. 0.2 × 0.15–0.25 cm, apex rounded; abaxial lip ca. 1.2 cm, 3-lobed, middle lobe oblong or obovate, 0.7–0.9 × 0.4–0.6 cm, lateral lobes oblong or obovate, closely equal, 0.6–0.7 × 0.3–0.5 cm, apex rounded. Stamens 4, coherent in 2 pairs, included, adaxial stamens ca. 7 mm, adnate to corolla tube ca. 4 mm from base, abaxial stamens ca. 8 mm, adnate to corolla tube ca. 6 mm from base; filaments white, adaxial ca. 0.7 cm, abaxial ca. 0.8 cm; anthers ca. 1 mm long, oblong, basifixed, dehiscing longitudinally; staminode 1, ca. 0.4 cm long, completely adnate to tube. Pistil included, ca. 0.7 cm long, glabrous; ovary columned, ca. 0.3 cm long, 2-loculed, glabrous; style ca. 0.3 cm, glabrous; stigmas 1, retuse; disc ring-like, ca. 0.1 cm high, margin dentate. Capsule straight, oblong, 2.0–2.5 cm long, existing style ca. 0.2 cm. Seeds not seen.

#### Distribution, habitat and phenology.

This species is only distributed in Yongde, Yunnan Province and grows on the rocks in montane forests. Flowering is September–October and fruiting is September–November.

#### Etymology.

The epithet “fulva” is named after the golden-brown villi on the plants.

#### Conservation status.

There is only a population with ca. 200 mature individuals and ca. 20, 000 m^2^ area (400 m × 500 m) from the type locality outside the nature reserve. Due to the vicinity of the local villages, the population is extremely affected by walnut planting. According to the IUCN Red List Categories and Criteria, the new species is assessed as “Critically Endangered (CR)” (B1ab(iii)+C2a(ii)). (IUCN 2012)

#### Note.

*Oreocharis fulva* is different from the other species in the tetrandrous and yellow-flowered group of *Oreocharis* s.l. and unique in its expanding corolla lips and narrow and short corolla tubes, with slight similarity to *Oreocharis georgei* in the morphology and texture of leaves (Fig. 3A–F, Table 2). Additionally, the new species and its similar species are respectively distributed in Southwest Yunnan (Lincang District) and Northwest Yunnan (Dali Bai Autonomous Prefecture and Lijiang District) without overlapping geographical distribution (Fig. 1, Li and Wang 2005).

**Table 2.** Differences in characters between *Oreocharis fulva* and *O. georgei* in Gesneriaceae.

Characters	*O. fulva* sp.nov.	*O. georgei*
leaf blade	elliptic	narrowly ovate to elliptic or narrowly obovate
adaxial surface of leaf	glabrescent	pubescent
abaxial surface of leaf	golden-brown villous between veins	glabrescent between veins
leaf base	shallowly cordate	narrowly cuneate
leaf apex	obtuse	acute to obtuse or acuminate
calyx lobe	ca. 8 mm long	2–4 mm long
corolla tube	not constricted at throat	constricted at throat
corolla lip	adaxial lip 3–4 mm, abaxial lip ca. 12 mm long	adaxial lip 2–3 mm long, abaxial lip 5–6 mm long
stamens coherent	2 pairs	separated
elevation	ca. 2000 m	2300–3400 m
distribution	SW Yunnan, China	NW Yunnan and SW Sichuan, China

### Oreocharis lacerata

Taxon classificationPlantaeLamialesGesneriaceae

W.H.Chen & Y.M.Shui sp. nov.

E3D6C95F-F02E-5A2A-A641-6DFA1F7D186F

urn:lsid:ipni.org:names:77211185-1

Fig. 6

#### Diagnosis.

The new species is similar to *Oreocharis concava* (Craib) Mich.Möller & A.Weber, but different in leaf margin lacerate (*vs.* unlobed), corolla strigose or glandular outside (*vs.* glabrous), abaxial lip much larger than the adaxial corolla lip (*vs.* nearly equal).

**Figure 6.**
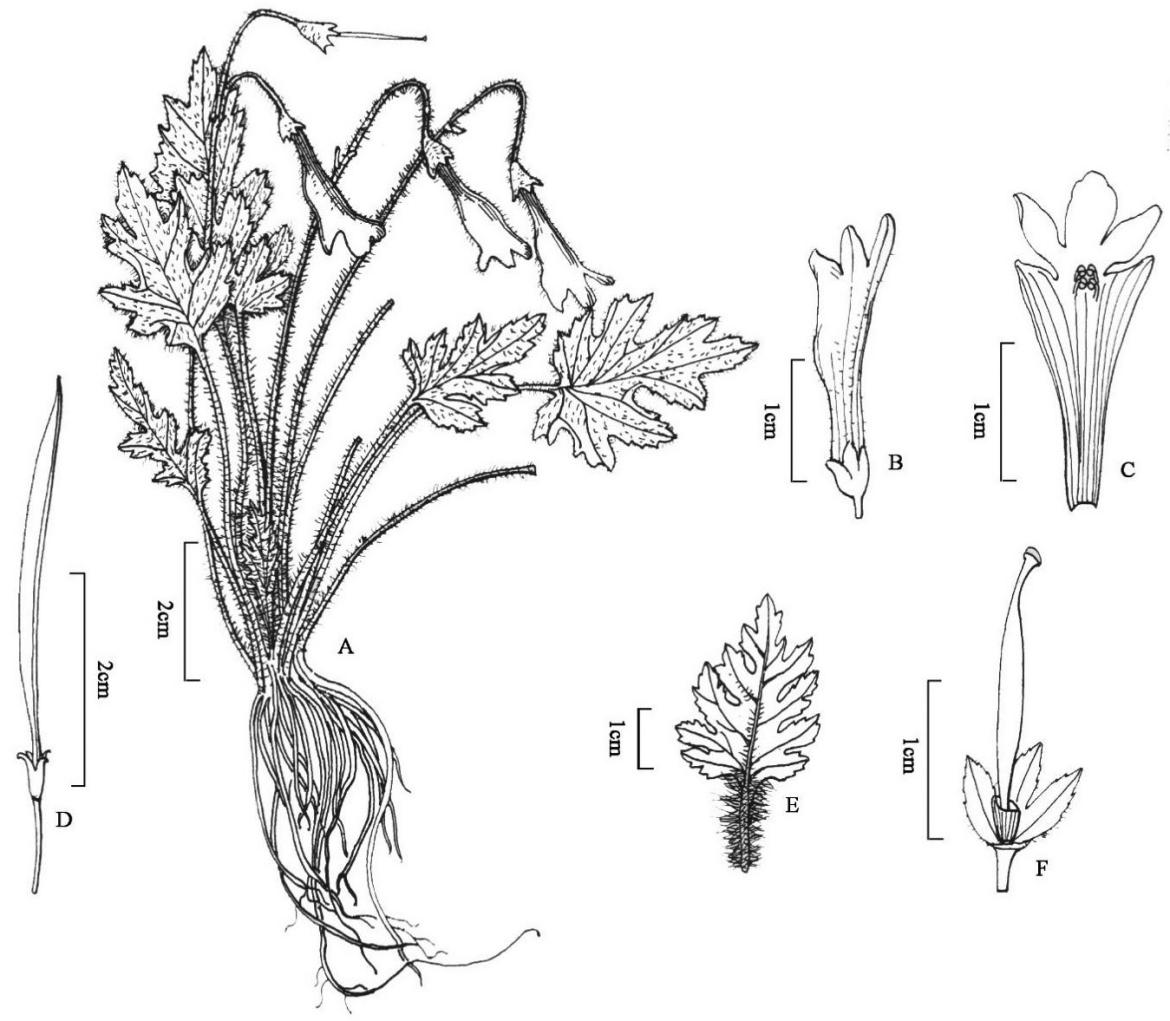
*Oreocharis lacerata* W.H.Chen & Y.M.Shui, sp. nov. **A** plant **B** flower **C** opened corolla showing stamens **D** fruit **E** abaxially leaf blade **F** pistil showing calyx, ovary, stigma and disc. (Drawn by Ling Wang from holotype).

#### Type.

CHINA. Yunnan Province: Lincang district, Yongde county, Womulong xiang, Ganhe village, Daliang Mt., on rocks along slope, alt. 2700 m, riverside, rare, 5 August 2003, *Zi S. S. 208* (holotype, KUN; isotype, PE).

#### Description.

Herbs perennial, stemless, rhizomatous. Leaves basal. Petiole 4–6 cm, covered with long golden multi-articulate hairs; leaf blade broadly lanceolate or elliptic, 3–4 × 2–2.5 cm, adaxially sparely puberulent, abaxially setal and with long golden multi-articulate hairs on ribs, base cordate, margin lacerate, lobes oblong and serrate, apex obtuse; lateral veins 3–4 pairs on each side of midrib. Inflorescences axillary, with many flowers. Peduncles 8–10 cm, golden-brown villous; bracts 2, ovate, ca. 2 × 1 cm. Calyx ca. 0.5 cm, 5-sect from middle, lobes triangular, 0.1–0.2 × ca. 0.15 cm, glabrous, apex acute, margin crenate. Corolla yellow, campanulate-cylindrical, 2.3–2.4 cm long, outside sparely multi-articulate strigose, inside glabrous; tube 1.8–1.9 cm long, ca. 1.5 mm in diam. at base and ca. 5 mm in diam. at throat, inflated above the middle; limb 2-lipped, adaxial lip 2-lobed, lobes semi-rounded, 3.1–3.4 × 0.3–3.2 mm, apex obtuse; abaxial lip explanate and 3-lobed, middle lobes oblong, 5–6 × 2.0–2.6 mm, glabrous, apex obtuse. Stamens 4, coherent in 2 pairs, included, adaxial stamens ca. 1.2 cm, adnate at the throat of corolla, abaxial stamens ca. 1.7 cm, adnate to corolla tube ca. 1.2 cm from base; filaments white, glabrous; anthers ca. 0.1 cm, oblong, basifixed, dehiscing longitudinally; staminode 1, ca. 0.1 cm long. Pistil included, ca. 1.2 cm long, glabrous; ovary columned, glabrous, ca. 0.7 cm long, 2-loculed; style glabrous, ca. 0.2 cm; stigma 1, undivided, oblate; disc ring-like, ca. 0.15 cm high, margin dentate. Capsule straight, oblong, 2–3.8 cm long, existing style ca. 0.2 cm. Seeds ovate, 0.6–0.63 × 0.21–0.24 mm.

#### Distribution, habitat and phenology.

This species is distributed in Yongde county, Yunnan Province, SW China. Flowering is August and fruiting is September–November.

#### Etymology.

The species is named after the lacerate leaves.

#### Conservation status.

The new species has been observed only from the type locality. The preliminary observation revealed that there are 300 mature individuals and ca. 600 m^2^ (20 m × 30 m) area nearby the summit in the core area of the nature reserve, almost never to be affected by the activity of the local people. According to the IUCN Red List Categories and Criteria, the new species is hereby assessed as “Vulnerable (VU)”(D). (IUCN 2012)

#### Additional specimens examined (paratypes).

CHINA. Yunnan province: Lincang district, Yongde county, Wumulong community, Ganhe village, Daliang Mt., 99°38'58"E, 24°08'56"N, on rocks in shrubs, elev. 2,902 m, flowers yellow, common, 11 August 2003, in flower, *Zi S.S. 261* (KUN, PE); the same locality, elev. 2,900 m, rare,16 September 2013, *Li Yong-Liang YDDXS 1137* (KUN).

#### Note.

*Oreocharis lacerata* is more similar to *O. concava* in the morphology of flowers than other species in the group with tetrandrous and yellow flowers, but differs mainly in the lacerate leaf margin (*vs.* unlobed) and obviously longer inferior lip of corolla than the superior lip (*vs.* equal between the two lips of corolla) (Fig. 3G–M, Table 3). With its pinnatilobate leaves, the new species is slightly more similar to *Oreocharis eximium* in the yellow-flowered group in *Oreocharis* and to *O. pinnatilobata* (K.Y.Pan) Mich.Möller & A.Weber in the purple-flowered group, but differs considerably in the morphology of flowers and fruits (Wang et al. 1990, 1998, Li 1991, Li and Wang 2005, Möller et al. 2011).

**Table 3.** Differences between *Oreocharis lacerata* and *O. concava* in Gesneriaceae.

Characters	*O. lacerata* sp.nov.	*O. concava*
leaf blade	margin lacerate, base cordate	margin un-lobed, base cuneate
adaxial surface of leaf	sparely puberulent	densely white puberulent and sparsely brown villous
corolla	campanulate-cylindrical, outside sparely multi-articulate strigose	cylindrical, outside densely pubescent
corolla tube	ca. 1.5 mm in diam. at base and ca. 5 mm in diam. at throat, inflated above the middle	1.7–2.2 mm in diam. at base and ca. 2.0–2.6 mm in diam. at throat, slightly inflated above the middle
adaxial corolla lip	apex obtuse, 2-lobed, lobes semi-rounded, 3.1–3.4 × 0.3–3.2 mm	apex acute, emarginate to undivided, lobes less than 1 mm or lacking
elevation	2700–2902 m	3100–3600 m
distribution	SW Yunnan, China	NW Yunnan, China

### Oreocharis wenshanensis

Taxon classificationPlantaeLamialesGesneriaceae

W.H.Chen & Y.M.Shui sp. nov.

D3703CFF-4B33-51DE-AC7C-E743C425E6B2

urn:lsid:ipni.org:names:77211186-1

Fig. 7

#### Diagnosis.

The new species is most similar in leaves to *Oreocharis concava* (Craib) Mich. Möller & A.Weber, but differs in broadly ovate leaf blade (*vs.* oblong-ovate), remotely pubescent adaxial leaf surface (*vs.* villous), calyx margin crenate (*vs.* irregularly dentate), the shorter corolla (1.5–1.6 cm long *vs.* 2.2–2.8 cm), adaxial corolla lip bilobed (*vs.* emarginate to undivided) and disc subentire (*vs.* 5-lobed).

**Figure 7.**
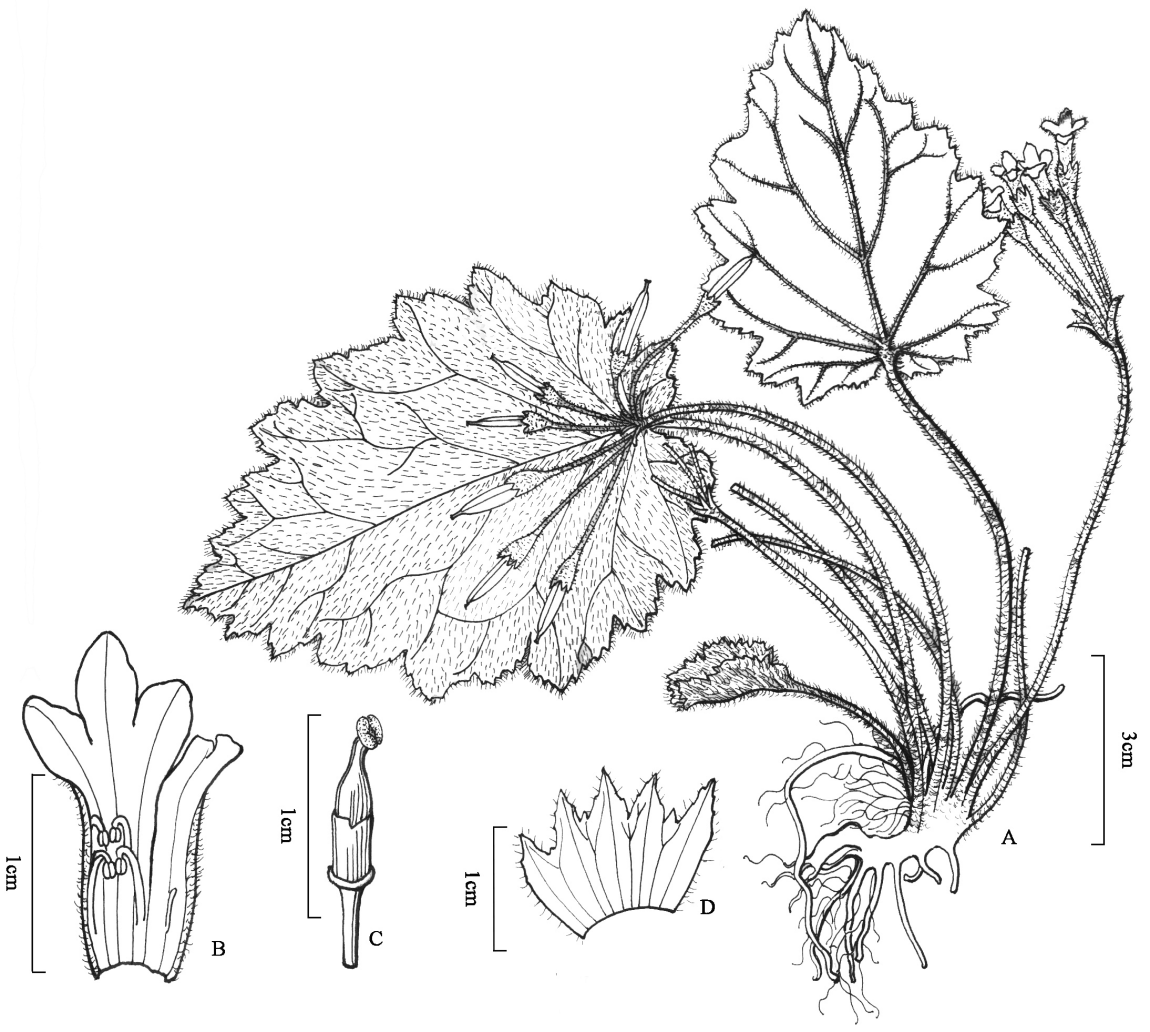
*Oreocharis wenshanensis* W.H.Chen & Y.M.Shui, sp. nov. **A** plant **B** opened corolla showing stamens and staminode **C** pistil showing ovary, disc and stigma **D** calyx. (Drawn by Ling Wang from holotype).

#### Type.

CHINA. Yunnan Province: Wenshan county, Bozu Mt., 23°21'1.41"N, 103°55'6.20"E, in dense forests, elev. 2,700 m, 27 July 1993, in flower, *Shui Y.M. 3126* (holotype, KUN!; isotype, PE!).

#### Description.

Herb perennial, stemless, rhizomatous. Leaves many, basal; petiole 5–9 cm, densely pubescent; leaf blade broadly ovate, chartaceous, 5.0–9.0 × 3.7–7.0 cm, adaxially sparsely pubescent and abaxially along veins, base cordate, apex acuminate, margin biserrate, lateral veins 4–5 on each side of midrib, indistinct adaxially and distinct abaxially. Inflorescences axillary, peduncle 6–10 cm, densely pubescent; bracts narrowly oblong, ca. 0.9 × 0.2 cm, adaxially glabrous, abaxially sparsely pubescent, apex acuminate, margin serrate above middle and entire below middle. Inflorescences axillary, densely cymose. Peduncles 5–7 cm, pubescent; Pedicels 2.0–2.8 cm, pubescent. Calyx 6–7 mm, 5-sect from 2/3; segments equal, triangular, ca. 3 × 2 mm, adaxially glabrous, abaxially pubescent, margin serratulate. Corolla yellow, 1.5–1.6 cm long, outside pubescent and inside glabrous; tube cylindrical, gradually slightly widening from middle of tube, 0.7–1.0 cm long, ca. 0.3 cm in diam., throat not constricted; limb 2-lipped; adaxial lip smaller, ca. 3 mm long, 2-lobed, lobes oblong, apex obtuse, 1–1.5 × ca. 1.5 mm; abaxial lip larger, 4–5 mm long, 3-lobed, lobes oblong, apex acute, central lobe 4–5 × ca. 3 mm, lateral lobes ca. 3 × 3 mm. Stamens 4, coherent in 2 pairs, included, adaxial stamens ca. 2 mm, adnate to corolla tube ca. 3 mm from base, abaxial stamens ca. 4 mm, adnate to corolla tube ca. 3 mm from base; filaments tender, glabrous; anthers basifixed, oblong, 2-loculed, dehiscing longitudinally; staminode 1, 1–2 mm, adnate to corolla tube 2–3 mm from base. Disc ring-like, 1–2 mm, subentire. Pistil 2.5–6 mm, glabrous; ovary oblong, glabrous, 1–4 mm, 1-loculed; style glabrous, 1.5–2 mm; stigma 1, top retuse. Capsule straight, oblong, 1.3–1.8 cm, existing style ca. 0.2 cm. Seeds not seen.

#### Distribution, habitat and phenology.

The new species only grows in the montane forest in Wenshan county, Yunnan Province of China. Flowering is July–September; and fruiting is October–January the following year.

#### Etymology.

The species is named after the type locality of the new species.

#### Conservation status.

Currently, the new species has been observed only from the type locality. The more than two years observation revealed that there are ca. 50 mature individuals and ca. 300 m^2^ (10 m × 30 m) area nearby the summit in the core area of the nature reserve, similarly to the above species (*O. lacerata*). According to the IUCN Red List Categories and Criteria, the new species is hereby assessed as “Critically Endangered (CR)” (D1+D2). (IUCN 2012)

#### Additional specimens examined (paratypes).

CHINA. Yunnan Province: Wenshan county, Laojun Mt., on rocks in forests, 23°21'1.41"N, 103°55'6.20"E, 31 August 2012, in flower, *Shui Y.M., He D.M. et al. B2012-099* (KUN); the same locality, on rocks in bamboo, 23°21'1.45"N, 103°55'6.24"E, 24 June 2013, *Shui Y.M. & Xiao B. B2013-100C* (KUN).

#### Note.

*Oreocharis wenshanensis* was collected first in 1993 by Y. M. Shui in Wenshan county, SE Yunnan, China (Fig. 2G–K). In 2005, the new species was wrongly identified as Oreocharis aurea Dunn var. cordato-ovata (C.Y. Wu ex H.W. Li) K.Y. Pan, A.L. Weitzman & L.E. Skog, based on the photo in Li and Wang (2005) possibly because of their similar locality in SE Yunnan. However, the latter species endemically grows in the limestone forests in Xichou county, the neighbouring county of Wenshan county, a very different habitat from *Oreocharis wenshanensis*. Furthermore, the new species we proposed can be easily distinguished from Oreocharis aurea Dunn var. cordato-ovata by its broadly ovate blade, smaller corolla without contracted throat and bilobed adaxial corolla lips (Li and Wang 2005). In fact, due to its 2700 m elevation, it is morphologically more similar to *O. concava* from NW Yunnan with 3100–3400 m elevation (Fig. 7, Table 4). After more than 20 years of observation, with the support of local staff of the Nature Reserve, we made a long-term observation from 1993 to 2018 and confirmed its taxonomic novelty.

**Table 4.** Differences between *Oreocharis wenshanensis* and *O. concava* in Gesneriaceae.

Characters	*O. wenshanensis* sp.nov.	*O. concava*
leaf blade	broadly ovate	oblong-ovate
adaxial leaf surface	remotely pubescent	villous
Inflorescences	densely cymose, flowers fascicular	sparely cymose, flowers sparse
corolla	1.5–1.6 cm long	2.2–2.8 cm long
calyx	ca. 3 mm, margin irregularly dentate	7–10 mm, margin crenate
corolla lip	adaxial lips slightly smaller than abaxial lips, adaxial ca. 3 mm long, abaxial lip 4–5 mm long	adaxial lips much smaller than abaxial lips, adaxial 1.5–3 mm long; abaxial ca. 7 mm long
adaxial lips	2-lobed	emarginate to undivided
disc	subentire	5-lobed
elevation	2700 m	3100–3400 m
distribution	SE Yunnan, China	NW Yunnan, China

## Supplementary Material

XML Treatment for Oreocharis eriocarpa

XML Treatment for Oreocharis fulva

XML Treatment for Oreocharis lacerata

XML Treatment for Oreocharis wenshanensis
